# Facial soft tissue response to maxillo-mandibular advancement in obstructive sleep apnea syndrome patients

**DOI:** 10.1186/s13005-017-0149-x

**Published:** 2017-06-23

**Authors:** Julio Cifuentes, Christian Teuber, Alfredo Gantz, Ariel Barrera, Gholamreza Danesh, Nicolas Yanine, Carsten Lippold

**Affiliations:** 1 Department of Oral and Maxillofacial Surgery, Clinica Alemana, Av Vitacura 5951, Vitacura, Santiago, Chile; 2 Department of Oral and Maxillofacial Surgery, Pontificia Universidad Católica de Chile, Av Libertador Bernardo O’Higgins 340, Santiago, Chile; 3 Department of Orthodontics, Faculty of Health, University Witten/Herdecke, Alfred-Herrhausen-Strasse 44, 58455 Witten, Germany; 4 Department of Orthodontics, Universitätsklinikum Münster, Albert-Schweitzer-Campus 1, Gebäude W30, Waldeyerstraße 30, 48149 Münster, Germany

**Keywords:** Obstructive sleep apnoea syndrome, Maxillo-Mandibular Advancement, Facial soft tissue change

## Abstract

**Background:**

Facial profile soft tissue changes after orthognathic surgery are crucial for surgery success. This retrospective study evaluated soft tissue changes after maxillo-mandibular Advancement and counter clockwise rotation surgery in obstructive sleep apnea syndrome patients.

**Methods:**

Thirty-seven obstructive sleep apnea syndrome patients (30 male, 7 female, mean age 35.8 years) whose underwent maxillo-mandibular-advancement and counter clockwise rotation surgery were studied after two intervals of time, presurgical, postsurgical and follow up (1–6 months and 1–5 years) using Dolphing Imaging Software. The soft tissue changes that were evaluated included Glabela, nasal projection, Subnasale, superior incisor, superior lip, inferior incisor, inferior lip, soft tissue B’ point and soft tissue Pogonion. Points were measured from true vertical line on the horizontal plane according to Arnett soft tissue profile analysis. Wilcoxon test was applied for testing differences between T0 (pre surgical), T1 (1–6 months postsurgical) and T2 (1–5 years postsurgical).

**Results:**

Cephalometric points changed to more aesthetic parameters. The largest advancements took place in the mandible, due to patients’ anatomic characteristics and treatment planning, whose were measured at cephalometric points B′ (9,05 mm) and Pog’ (11,92 mm) at T0–T2. In all patients aesthetics goals were accomplished.

**Conclusion:**

This study showed that maxillo-mandibular advancement and counter clockwise rotation surgery is an effective treatment for OSAS, with good aesthetic results.

## Background

Obstructive sleep apnea syndrome (OSAS) is characterized by repetitive episodes of pharyngeal collapse with increased airflow resistance during sleep [1]. Risk factors include obesity, middle aged male gender, advanced age and an anatomically smaller upper-airway [1–7]. Up to 25% of adults represent signs and symptoms of OSAS, and approximately 10% of all adults have a moderate to severe level of OSAS [4, 5]. It is associated with higher rates of cardiovascular and cerebrovascular morbidity and mortality as well. Continuous positive airway pressure (CPAP) therapy has been considered the reference standard treatment for OSAS. However, despite the potential success of CPAP, patient compliance represents a clear problem [8], causing them to seek surgical treatment alternatively. The goal of surgical treatment in OSAS is to enlarge the velo-oropharyngeal airway by anterior/lateral displacement of the soft tissues and musculature by maxillary, mandibular, and possibly genioglossus advancement [2]. From a medical point of view there are two major rationales for surgery that need to be well understood at the time of surgical MMA planning [2]. The first rationale is “behavioral derangement,” which is normally due to excessive daytime sleepiness (EDS). Symptoms may include snoring, apneas, morning headaches, fatigue, day sleepiness, memory loss, irritability or poor work performance [3–5]. The second rationale is “pathophysiologic derangement,” which is, in part, cardiorespiratory in nature. Three important well-known physiological processes are involved in OSAS that predispose to these risks: hypoxemia, negative intrathoracic pressure, and disequilibrium of the autonomic nervous system [1–5].

It has been reported in literature that MMA has a high rate of success: beginning in the early 1980s, several studies reported improvement in polysomnographic parameters in patients treated with isolated mandibular advancement surgery [4, 5, 9–11]. However, by the mid-1980s, combined MMA was preferred over mandibular osteotomy alone to treat OSAS patients with normal maxillo- mandibular relationship to preserve the maxillo-mandibular relations, and due to the recognition of the physiologic etiology of OSAS, which is often caused by concomitant mandibular and maxillary deficiencies [12, 13].

Holty and Guillaminault determined in their systematic review and meta-analysis that MMA is an effective treatment for OSAS [5]. The mean apnea hypopnea index (AHI) decreased from 63.9 (severe sleep apnea) to 9.5 (mild sleep apnea) with a pooled surgical success rate of 86%. They also determined univariate predictors for surgical success for young age, lower preoperative AHI, and greater degree of maxillary advancement. To determine the success of performing MMA, Pirkbauer et al.. [14] showed in a systematic review, that MMA therapy has good clinical results, even comparable to ventilation therapy for OSAS patients. Although MMA has been primarily recommended for patients with OSAS and significant maxillo-mandibular deficiency, it could also be advocated for the treatment of OSAS in patients with relatively mild maxillofacial abnormalities. It appeared that, despite the alteration of facial esthetics after MMA, more than 90% of the patients gave positive or neutral responses to their facial appearance after surgery [15].

As a large proportion of OSAS sufferers are mature adult males [4, 15], a number of patients requiring MMA surgery will present with a face showing signs of ageing, in particular skeletal atrophy, a sagging tip of the nose and hollow cheeks. Because MMA brings forward the skeletal structures of the midface and lower face complex and strains cutaneous soft tissues, the procedure can rejuvenate the patient’s appearance. Facial changes resulting from MMA are generally well received [14, 15]. Nevertheless, a number of patients find they are less attractive following the procedure. Li et al. found that 10% of their patients thought that they were less attractive [15]. These negative effects, which have also been described by other surgical teams, are nonetheless considered to be of secondary importance by patients compared with the benefits achieved by the surgery [1–5, 9–12]. More research is required into these “aesthetic failures” as a number of patients could refuse surgery to avoid the risk of facial deformity. This is especially true for young subjects with full faces or for women with finer soft tissues, which would not conceal their skeletal contours.

It has been demonstrated that during MMA, the soft tissues follow skeletal displacement to a large extent in the anteroposterior dimension [16–18]. To reduce the convexity of the upper lip, some surgeons systematically incorporate counter-clockwise rotation into the maxillo-mandibular complex during the advancement; but in our surgical team it is used due to the advancement of the mandible.

While soft tissue changes after orthognathic surgery have been studied for many years, little is known about the changes in facial appearance after MMA in patients with OSAS.

In our present retrospective study we performed a cephalometric analysis of soft tissue changes in a typical group of OSAS patients. The hypothesis in this study, was that there are significant differences between the measured values at T0 and T1 and T0 and T2.

## Methods

**Fig. 1:**
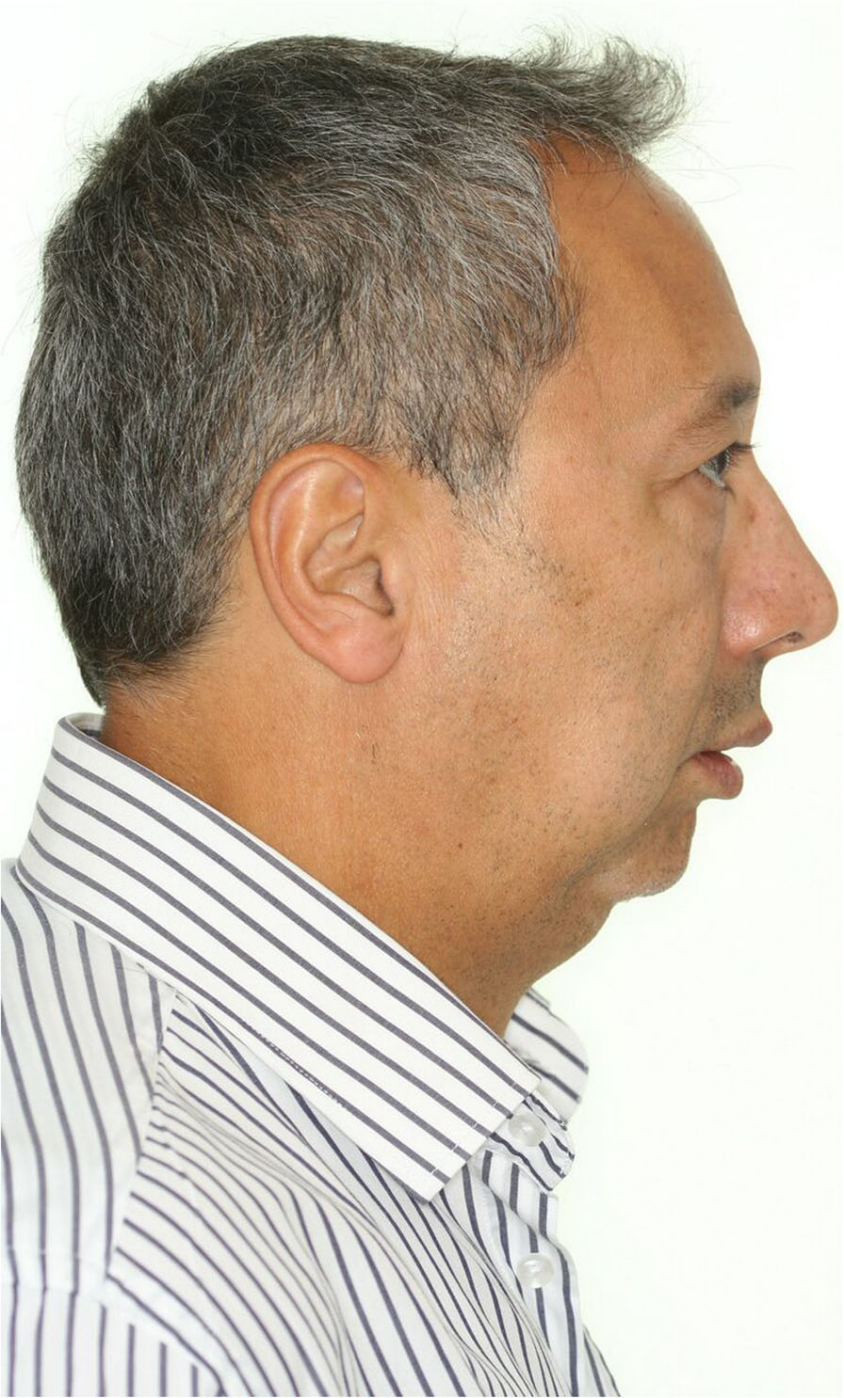
Extraoral photograph of a sample patient *before* maxillo-mandibular advancement surgery

**Fig. 2:**
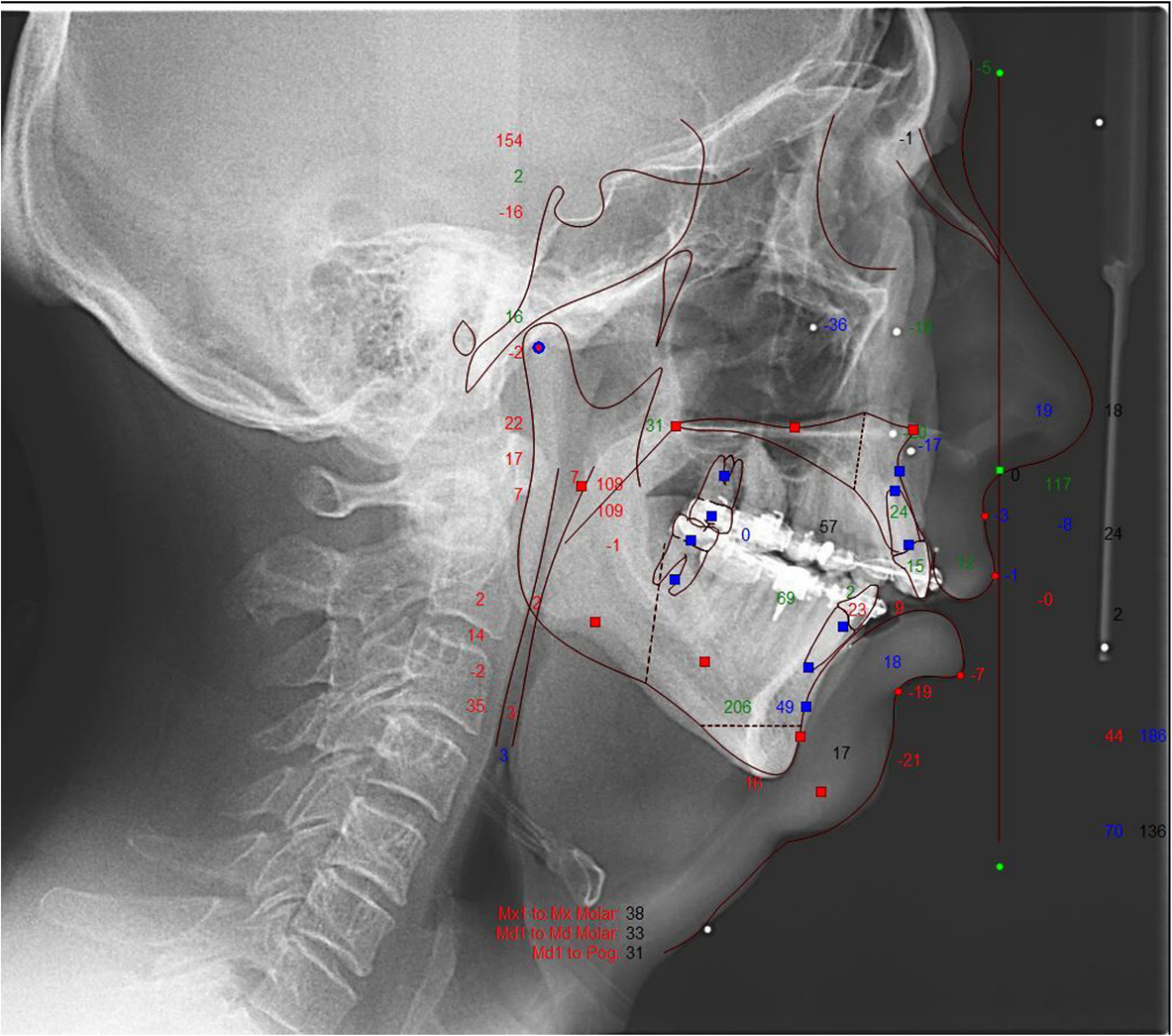
Lateral cephalograph of a sample patient *before* maxillo-mandibular advancement surgery

**Fig. 3:**
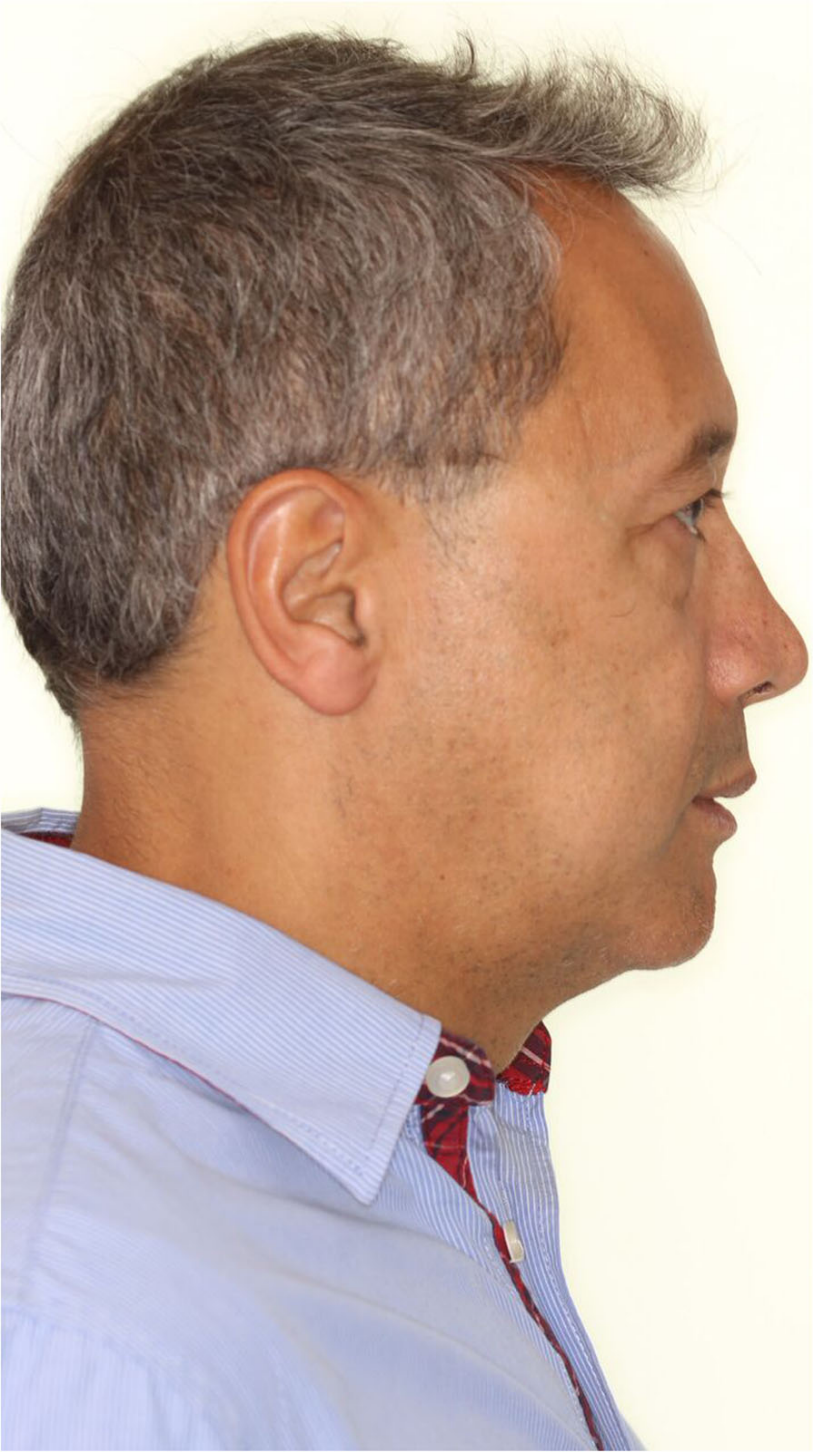
Extraoral photograph of a sample patient *after* maxillo-mandibular advancement surgery

**Fig. 4:**
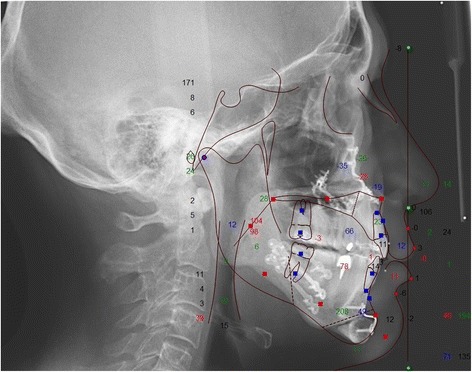
Lateral cephalograph of a sample patient *after* maxillo-mandibular advancement surgery

The study sample was compounded of 37 patients (30 males and 7 females) matching the inclusion and exclusion criteria of this retrospective analysis. The patients mean age were 35.8 years. At time of surgery, the youngest patient was 21.1 years old while the oldest patient was 56.2 years old. All OSAS patients were operated on by the same surgical team at the oral and maxillofacial unit (Clínica Alemana, Santiago, Chile) by maxillo-mandibular advancement and counter clockwise (CCW) rotation (Figs. 1, 2, 3 and 4).

All patients were treated in the same way with regard to preoperative, perioperative and postoperative care. Under general anesthesia a Le Fort I and segmental maxillary osteotomy was performed in addition to a bilateral sagittal osteotomy of the mandibular ramus and a mental osteotomy. The latter osteotomies were performed to allow maxillo-mandibular and chin advancement. Rigid internal fixation was performed with titanium plates (KLS – Martin and Osteomed). Four “L” miniplates were used in the maxilla, and four “straight” miniplates were used in the bilateral sagittal osteotomy (2.0 mm miniplates, with four 2.0 mm monocortical screws), to give more stability to the mandibular advancement with CCW Rotation. One genioplasty plate, “double Y shape,” was used in chin advancement. The movements of the double jaw orthognathic surgery, in addition to chin advancement, were mainly counterclockwise to maxilla-mandibular advancement.

Inclusion criteria were all patients diagnosed with OSAS, skeletal class II, >18 years, who were treated with MMA and CCW rotation. In all patients with these skeletal characteristics we performed MMA with CCW rotation, because we can achieve an improved nasal airway, increased nasopharyngeal and oropharyngeal airway, normal chin projection and facial harmony.

Exclusion criteria included patients previously treated for maxillofacial deformities by other types of orthognathic surgery or orthodontics, facial trauma, systemic diseases and strokes not caused by OSAS.

**Fig. 5:**
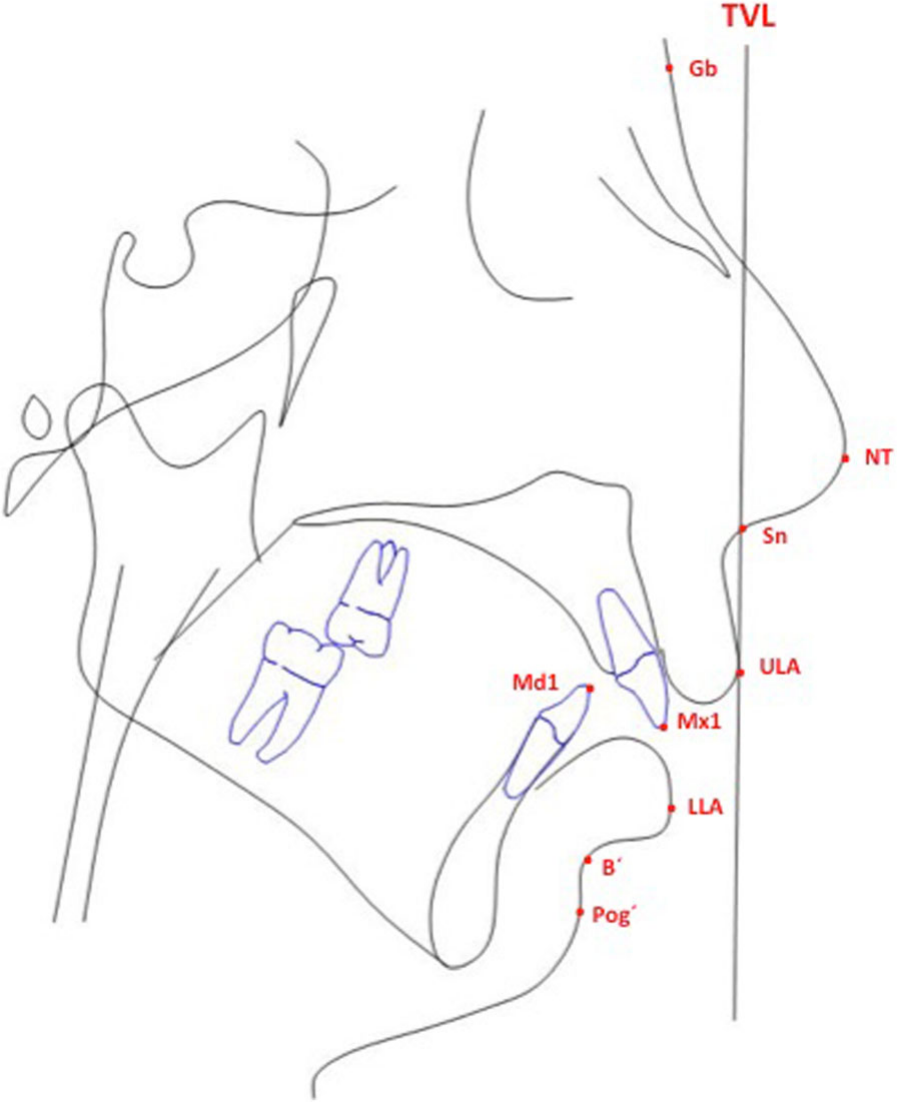
Facial lateral soft tissue cephalometric anaylsis according to Arnett et al. used in this study [11]

**Fig. 6:**
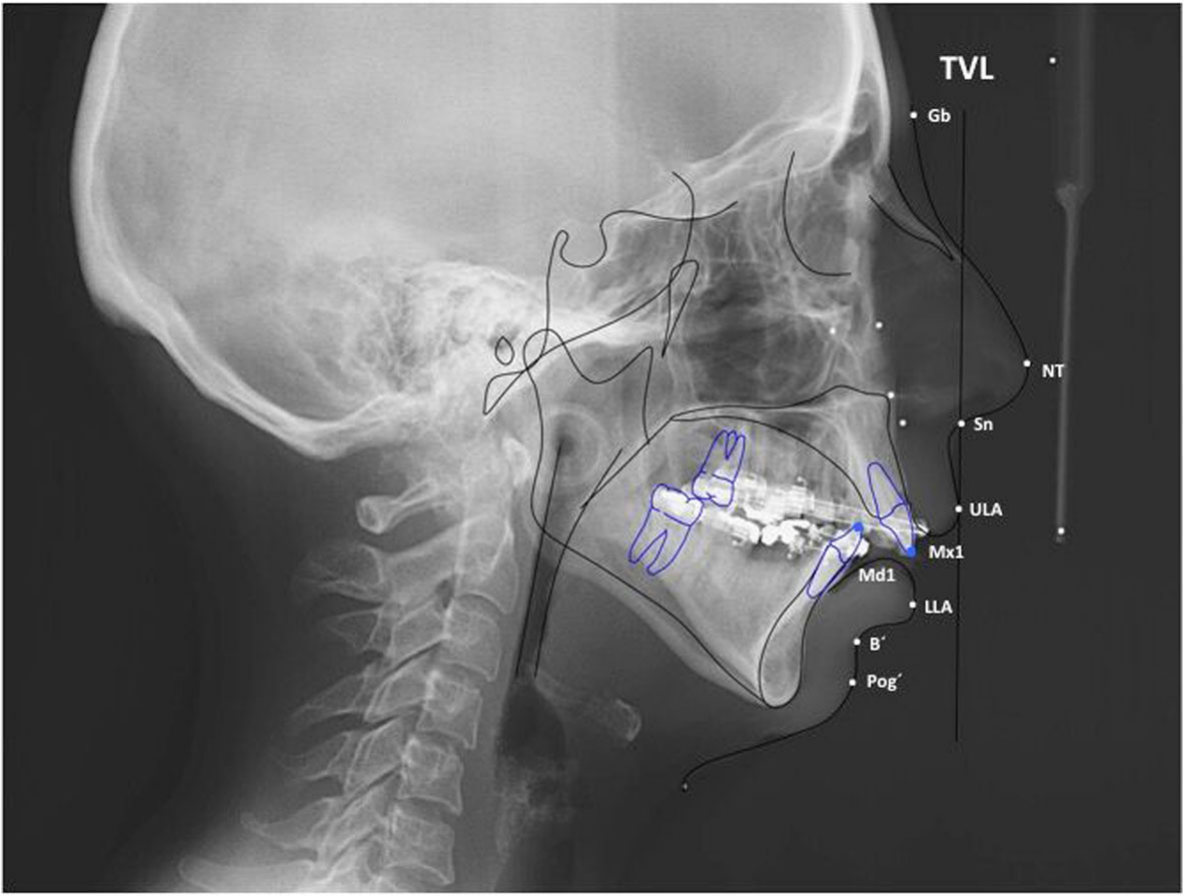
Sample of a cephamolmetric radiograph of the lateral soft tissue facial analysis

To assess the soft tissue changes of the surgical procedure cephalometric radiographs were analysed. Radiographs were taken at T0 presurgical, T1 at a mean of 5.2 weeks after surgery, and T2 at a mean of 2.1 years after surgery. They were performed with a standard length marker of 100.0 mm at natural head posture with passive lips as described in the method by Arnett et al. [11], using a Ortophos XG 3D (Sirona, Bensheim, Germany). The imaging proportion used was 1:1, which was digitalized with Dolphin Imaging Version 11.7 Premium (Chatsworth, CA, USA). Based on the cephalometric soft tissue points described by Arnett et al. [11], a cephalometric analysis was performed (Figs. 5 and 6). Nine points (in both hard and soft tissues) were selected in relation to the true vertical line (TVL), which is a perpendicular line that passes through the subnasal point: glabella, nasal projection, upper lip anterior, upper incisor, inferior lip, inferior incisor, soft tissue B point, soft tissue pogonion. The distance masurements were carried out between the reference points perpendicular to the TVL. Figure 5 shows the basic principle of this analysis, the corresponding points and the true vertical line. Figure 6 is an example of the analysis. Statistical data analysis was performed with SPSS Version 18.0 statistical software (SPSS Inc., Chicago, IL, USA). Kolmogorov–Smirnov test was used for testing the normal distribution. The samples showed no normal distribution at T0, T1 and at T2. The null hypothesis in this study was, that there are no significant differences between the measured values at T0 and T1 and T0 and T2. To assess differences between T0 and T1 and T0 and T2 the Wilcoxon signed-rank test was applied.

## Results

The results of the cephalometric analysis are shown in Table 1 as the mean and the standard deviation minimum and maximum values for T0, T1 and T2. The measurements were obtained in millimeters for each of the points evaluated in relation to the TVL for T0, T1 and T2. Positive values are for a position in front of the TVL, and negative values are for a posterior position. The statistical analysis with Wilcoxon test is shown in Table 2.

**Table 1:** Descriptive statistics of the patients sample (mean, SD, minimum, maximum)

		Descriptive Statistics				
		*N*	Mean	SD	Minimum	Maximum
T0	Gb	37	−6.811	3.2900	−15.0	−2.0
	NT	37	17.216	2.0835	11.0	21.0
	Sn	37	0.000	0.0000	0.0	0.0
	ULa	37	1.432	2.2304	−2.0	6.0
	Mx1	37	−12.865	7.2156	−20.0	25.0
	Mn1	37	−5.459	2.9401	−11.0	0.0
	LLa	37	−19.784	3.7575	−26.0	−13.0
	B′	37	−18.054	4.0411	−26.0	−6.0
	Pog’	37	−17.784	5.5183	−30.0	−4.0
T1	Gb	37	−10.000	3.4721	−20.0	−5.0
	NT	37	13.324	1.9868	8.0	18.0
	Sn	37	0.000	0.0000	0.0	0.0
	ULa	37	2.378	2.9660	−4.0	9.0
	Mx1	37	−17.216	3.2586	−25.0	−9.0
	Mn1	37	−3.703	2.7169	−10.0	2.0
	LLa	37	−19.946	3.4637	−29.0	−11.0
	B′	37	−11.216	3.7129	−20.0	0.0
	Pog’	37	−9.622	4.2645	−18.0	3.0
T2	Gb	37	−8.324	4.2495	−22.0	−1.0
	NT	37	15.243	2.0194	10.0	20.0
	Sn	37	0.000	0.0000	0.0	0.0
	ULa	37	1.649	2.7408	−4.0	6.0
	Mx1	37	−12.297	4.7950	−20.0	9.0
	Mn1	37	−2.757	3.2094	−10.0	4.0
	LLa	37	−15.595	3.1838	−22.0	−7.0
	B′	37	−9.000	4.2426	−18.0	−1.0
	Pog’	37	−5.865	4.5532	−17.0	3.0

Mean, standard deviation, minimum and maximum values in millimeters of the measurements for T0 (presurgical), T1 (1–6 months postsurgery) and T2 (6 months to 5 years after surgery) between the cephalometric points and the TVL

*Abbreviations*: *Gb* Glabella, *NT* Nasal Tip, *Sn* Subnasale, *ULa* Upper Lip Anterior, *Mx1* Upper Central Incisor Edge, *LLa* Lower Lip anterior, *Mn1* Lower Central Incisor Edge, *B′* Soft Tissue B-Point, *Pog’* Pogonion Molle

**Table 2:** Wilcoxon test at T0–T1 and T0–T2 for the patient sample

Statistics Wilcoxon test									
	Sn–Gb	Sn–NT	Sn–Sn	Sn–ULA	Sn–Mx1	Sn–LLA	Sn–Md1	Sn–B’	Sn–Pog’
T0–T1	−4.31	−5.24	0.000	−1.67	−4.49	−3.05	−0.20	−5.14	−5.15
*p*-value asymptotic significance (2-sides)	<0.001	<0.001	1.000	0.094	<0.001	0.002	0.837	<0.001	<0.001
T0–T2	−2.31	−4.35	0.000	−0.54	−2.15	−3.94	−4.78	−5.16	−5.24
*p*-value asymptotic significance (2-sides)	0.021	<0.001	1.000	0.586	0.032	<0.001	<0.001	<0.001	<0.001

The cephalometric points’ movement in millimeters (mm) and the proportion of movement (%) between the same points in T0 (presurgical), T1 (1–6 months postsurgery) and T2 (6 months to 5 years after surgery)

## Discussion

Skeletal changes upon orthognathic surgery determine the soft tissue facial profile, which is observed by many surgeons as one of the parameters of successful treatment [11–21]. The main objective of MMA for OSAS patients is to cure the disease and decrease AHI values. Although the OSAS can be cured, aesthetics takes a fundamental role in defining a surgical success.

This study assessed the changes in facial profile after skeletal movements observed in patients diagnosed with OSAS. The aim was to describe variations of soft tissues between presurgical (T0) and two postsurgical evaluations (T1 and T2).

The response of the facial soft tissues after orthognathic surgery may be influenced by various factors, such as presurgical, surgical and postsurgical variables. Presurgical variables may include concurrent soft tissue deformities, nasal deformities, a degree of mandibular retrusion, previous trauma or maxillofacial surgeries, and thickness, as well as length and tone of the soft tissue overlying the area. Surgical variables may include a degree of dissection, edema or hematoma formation, an amount of bony resection, an amount of graft procedures, amount and direction of MMA movement, and surgical closure techniques. Postsurgical variables may include degree of bony resorption, weight gain or loss after surgery, relapse of bone segments, resultant soft tissue scaring, postoperative infection, and soft tissue stability [17].

Presurgical variables cannot be controlled; however, surgical and most of the postsurgical variables may be controlled to produce predictable results. Facial tissue changes after MMA and CCW rotation appear to be stable at 6–8 months after surgery [6, 7, 16–18, 20–22]. The evaluation of facial aesthetics must be performed at least 6 months after surgery to obtain reliable results. In this study, we observed patients who had at least 12 months postsurgery.

Upper lip variations shown in Table 1 demonstrate that edema can increase the upper lip’s position from TVL up to 66% on the first 4–5 weeks postsurgery, but, as postsurgical time passes, so does the edema, and it finally stabilizes at 15.1% from the TVL, which is 0,22 mm. An inverse pattern occurs at nasal projection, where its position decreases −22.6% due to TVL association with edema. Nasal projection finally stabilizes at −11.5% from the TVL, which is −1.97 mm.

Resultant soft tissue scarring of the Le Fort I incision, and if nasolabial muscle reconstruction is performed or not, appear to affect the upper lip’s length and thickness. The V-Y closure procedure has been reported to decrease lip thickness by 2 mm. In the same context, an alar base cinch or nasalis muscle suture introduces a surgical variable, which is used to counteract widening of the alar base of the nose that occurs with advancement or superior repositioning of the maxilla [17, 23].

Previous studies have determined that translation or rotation of the maxilla did not lead to a significant volume increase of the nose [16]. However, anterior translation of the maxilla increases lip volume. When no anatomic reorientation of the nasolabial musculature is performed, thin lips follow the maxilla’s advancement to a greater degree than do thicker lips [6, 16, 17].

Mandibular soft tissues suffered the highest variations, as expected. All patients were skeletal class II, with a mean presurgical distance at the inferior lip of 5,46 mm, B’ of 18,05 and 17,78 mm for Pog’. Variations suffered at the inferior lip, B’ and Pog’ points from T0 to T2 were 2,70, 9,05 and 11,92 mm, respectively. There was a proportional variation of distance at T0–T1 produced by edema and TVL variation at the subnasal point of 32.2% at the inferior lip, 37.9% at B’ and 45.9% at Pog’. Tissues finally stabilized from T0 to T2 with variations of distance of 49,50% at the inferior lip, 50.2% at B’ and 67.0% at Pog’. Edema caused tissue enlargement, and it did not stabilize until 6–8 months after surgery.

Conley et al. [6] had similar results, with variations of distance at the inferior lip of 9.5 mm, B’ 11.6 mm, and Pog’ 15.1 mm. They determined that soft tissue changed by approximately 90% in relation to underlying dental skeletal movements. Several studies [5, 14] have assessed the mean advancement variations of the maxilla (7.4–8.7 mm) and the mandible (10.7–11.2 mm), but only a few studies have determined whether the patient truly accepted the aesthetic appearance. The studies of Li et al. [15, 19] revealed that soft tissue changes caused by MMA in their patient population appeared to result in a rejuvenation of the face. Ageing results in soft tissue descent with loss of lips and cheek prominence. MMA leads to skeletal expansion, which increases the soft tissue support with positive aesthetic effect, similar to the facelift procedure. Laxity of soft tissue and the facial thick envelope of the OSAS patients partially mask the effect of the skeletal advancement.

Arnett et al. [8, 24] have described in their studies normal aesthetic cephalometric guidelines, referring to the soft tissue cephalometric diagnosis. Therefore they measured patients in five different but interrelated areas. These areas were dentoskeletal factors, soft tissue structures, TVL Projection, Facial Length and Harmony Values. TVL projections are anteroposterior measurements of soft tissue and represent the sum of the dentoskeletal position plus the soft tissue thickness overlying that hard tissue landmark. The horizontal distance for each individual landmark, measured perpendicular to the TVL, is termed the landmark’s absolute value. Although subnasale will frequently be coincident with anteroposterior positioning of the TVL, they are not synonymous. For example, the TVL must be moved forward in cases of maxillary retrusion [8].

The results of the soft tissue cephalometric analysis of Arnett et al. [8] are quite similar to the results of our study. They described aesthetic results for different cephalometric points in females and males. Our study did not describe the cephalometric points by gender due to a smaller sample size and a small number of 7 female patients in comparison to 30 male patients. If we make a comparison between our results and Arnett et al. [8], they showed that the glabella was measured to be −8.5 mm in females and −8.0 mm in males; our study showed a measurement of −8.3 mm. The nasal projection measured at 16 mm for females and 17.4 mm for males; our study measured the nasal projection at 15.2 mm. The upper lip measurement was 3.7 mm for females and 3.3 mm for males; our study measured the upper lip to be 1.6 mm. The inferior lip measurement was 1.9 mm for females and 1.0 mm for males; our study measurement for the inferior lip was −2.8 mm. The B’ point measurement was −5.3 mm for females and −7.1 mm for males; our study measured the B’ to be −9 mm. In the same study the Pog’ was measured to be −2.6 mm for females and −3.5 mm for males; in our study we measured −5.9 mm. Our aesthetic cephalometric measurements from soft tissues analysis were not as described by Arnett et al. [8], but quite similar. However, we need to consider that these patients had skeletal class II relationships, obstructive sleep apnea syndrome as a severe disease, and an average measure of the B′point and the Pog’ of −18.1 mm and −17.8 mm, respectively.

According to the results of our study, there were no disproportionate postsurgery facial features, which otherwise could have affected social relationships and the quality of life.

## Conclusions

The results of our study confirm maxillo-mandibular advancement as a valid treatment for obstructive sleep apnea syndrome in patients with normal facial proportions and skelettal class II. An accurate understanding of the soft tissue response is necessary for treatment planning, prediction and patient education.

## Abbreviations

MMA: Maxillo- mandibular advancement
OSAS: Obstructive sleep apnea syndrome
TVL: True vertical line
